# Immunometabolic control by *Klebsiella pneumoniae*

**DOI:** 10.1097/IN9.0000000000000028

**Published:** 2023-07-24

**Authors:** Alice Prince, Tania Wong Fok Lung

**Affiliations:** 1 Department of Pediatrics, Columbia University, New York, NY, USA

**Keywords:** immunometabolism, metabolic reprogramming, *Klebsiella pneumoniae*, antimicrobial resistance, pulmonary infection, tolerance, resistance

## Abstract

*Klebsiella pneumoniae* is a common Gram-negative pathogen associated with community-acquired and healthcare-associated infections. Its ability to acquire genetic elements resulted in its rapid development of resistance to virtually all antimicrobial agents. Once infection is established, *K. pneumoniae* is able to evade the host immune response and perhaps more importantly, undergo metabolic rewiring to optimize its ability to maintain infection. *K. pneumoniae* lipopolysaccharide and capsular polysaccharide are central factors in the induction and evasion of immune clearance. Less well understood is the importance of immunometabolism, the intersection between cellular metabolism and immune function, in the host response to *K. pneumoniae* infection. Bacterial metabolism itself is perceived as a metabolic stress to the host, altering the microenvironment at the site of infection. In this review, we will discuss the metabolic responses induced by *K. pneumoniae*, particularly in response to stimulation with the metabolically active bacteria versus pathogen-associated molecular patterns alone, and their implications in shaping the nature of the immune response and the infection outcome. A better understanding of the immunometabolic response to *K. pneumoniae* may help identify new targets for therapeutic intervention in the treatment of multidrug-resistant bacterial infections.

## 1. Introduction

*Klebsiella pneumoniae* has long been appreciated as an opportunistic pathogen that causes both hospital- and community-acquired infections. Its organization into two distinct pathotypes, classical and hypervirulent, is mainly based on the individual patterns of clinical illness. While the hypervirulent *K. pneumoniae* strains cause fulminant and rapidly lethal infections in healthy individuals, the classical strains typically cause subacute and prolonged infections in immunocompromised patients. Nonetheless, the latter indolent infections frequently become fatal, as is commonly the case with ventilator-associated pneumonia ^[1,2]^. Classical *K. pneumoniae* strains develop resistance to multiple antimicrobial agents, including carbapenems and polymyxins. This is facilitated by their natural ability for chromosomal recombination as well as plasmid and phage acquisition ^[3]^. Multidrug-resistant (MDR) strains belonging to the sequence type (ST) 258 rapidly disseminated worldwide since their emergence in the early 2000s, earning a designation as global public health problem ^[1]^. To this day, MDR *K. pneumoniae* strains remain a major clinical problem.

Studies on comparative genomics and antimicrobial resistance mechanisms have significantly improved our understanding of the evolution and biology of *K. pneumoniae* ^[3–7]^. How *K. pneumoniae* evades the host immune response through the subversion of signaling pathways has also been the subject of intensive investigation ^[8]^. Less well appreciated are the very substantial effects on host metabolism that are induced during the course of *K. pneumoniae* infection. While some of the metabolic pathways activated during the immune response to bacterial infection are associated with proinflammatory responses, others are anti-inflammatory and immunosuppressive. The induction of host glycolysis by bacterial lipopolysaccharide (LPS) and the accumulation of specific inflammatory metabolites such as succinate polarize macrophages into the proinflammatory M1-like phenotype to promote bacterial clearance ^[9,10]^. However, an exaggerated proinflammatory response can activate damaging pathology in the host, sepsis, and death. Bacteria that instead promote the induction of anti- inflammatory responses persist to cause intractable infection.

There is increasing evidence that MDR *K. pneumoniae* isolates typical of healthcare-associated pneumonia, elicit an immunosuppressive response in the airway. This immune response is dominated by the early accumulation of immature myeloid cells and the host anti-inflammatory metabolite itaconate ^[11,12]^. Thus, the immune response to classical MDR *K. pneumoniae* is in contrast to the brisk inflammatory response stimulated by hypervirulent *K. pneumoniae* that is associated with immunopathology. The use of a mouse pneumonia model, despite its limitations, has helped to illustrate the importance of the host immunometabolic response in determining the infection outcome ^[12]^. In this review, we will focus upon the metabolic responses that are activated by healthcare-associated *K. pneumoniae* and how they drive an immune response tolerant of infection and contribute to its success as a pulmonary pathogen.

## 2. *K. pneumoniae* pathotypes

**Figure 1.**
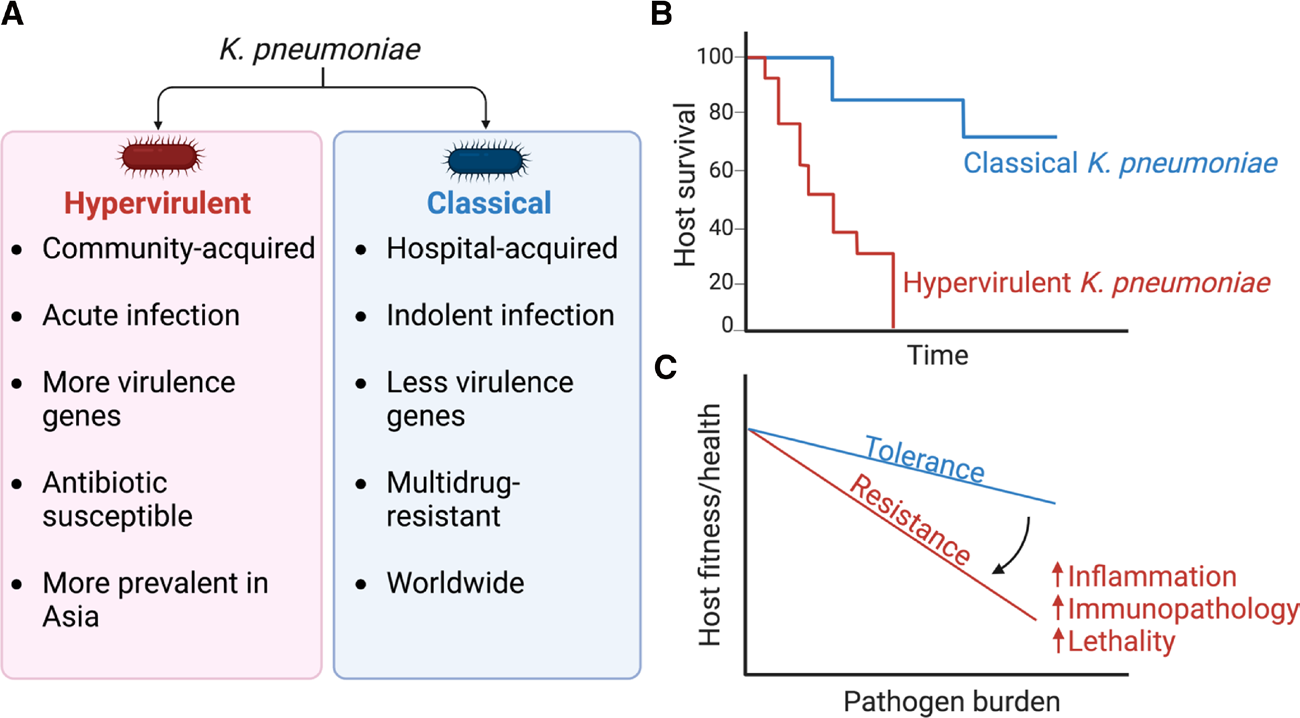
***K. pneumoniae* pathotypes induce different host immune defense strategies.** (A) Several factors determine the classification of *K. pneumoniae* into pathotypes. (B) Schematic diagram illustrates the outcome of infection by classical versus hypervirulent *K. pneumoniae*. (C) Diagram illustrates different host defense strategies during infection with bacterial pathogens. The prioritization of host fitness over pathogen control in response to classical *K. pneumoniae* infection is in line with a tolerant rather than a resistant host immune response to infection.

The classification of *K. pneumoniae* into the hypervirulent or classical pathotypes rests largely on the clinical patterns of illness associated with each. These designations have been further defined by differences in the accessory genome and the distinctive epidemiology of the infections (Figure 1A). The criteria for what constitute hypervirulence can be confusing particularly given the many changes in the literature over time. *K. pneumoniae* strains that are currently defined as hypervirulent cause acute community-acquired infections such as pyogenic liver abscesses that metastasize to distant sites including the lung and the central nervous system. These strains are generally antibiotic susceptible and display enhanced virulence owing to a multitude of horizontally acquired genetic elements ^[13]^. The acquired genes encode virulence determinants such as siderophores and an antiphagocytic capsule that resists complement-associated clearance and phagocytosis ^[13]^. The production of a thick capsule was previously thought to be associated with the hypermucoviscous phenotype of hypervirulent *K. pneumoniae*. These are now known to be distinct phenotypes, which are not necessarily features of all hypervirulent strains ^[14]^. Although hypervirulent *K. pneumoniae* strains were first recognized in the 1980s in Southeast Asia, they are likely to have predated this time, particularly based on clinical observations. Historically, upper lobe necrotizing pneumonias (also called Friedlander’s or currant-jelly pneumonias) associated with severe pathology, and death were eventually recognized to be caused by *K. pneumoniae*, presumably hypervirulent strains. The STs most commonly associated with hypervirulence include ST23, ST65 and ST86.

Over the course of the past two decades, other *K. pneumoniae* STs have become prominent worldwide, especially in healthcare settings. These classical strains are often multiply antibiotic resistant, particularly the ST258, but lack many virulence factors. Although these strains cause subacute infections ^[15]^, they persist in the host, leading to substantial morbidity and mortality, and their inclusion as a pathogen of concern by the Centers for Disease Control and Prevention (CDC) and the World Health Organization (WHO) ^[1]^. These more subacute infections may be caused even by strains that are more antibiotic susceptible ^[16,17]^, indicating that other bacterial factors are important in the pathogenesis of classical *K. pneumoniae*. The association of these classical strains with outbreaks in intensive care units (ICUs) worldwide have spurred investigation into their modes of pathogenicity, particularly as compared to the hypervirulent strains ^[18,19]^. The remarkable ability of *K. pneumoniae* to evade immune defenses has been recently reviewed ^[8]^.

Host signaling pathways that contribute to an effective airway immune response against *K. pneumoniae* have been thoroughly investigated ^[20]^. IL-17 is especially important as it protects from both *K. pneumoniae* pathotypes. Overexpression of IL-17 in the murine airway promotes the clearance of hypervirulent *K. pneumoniae* and *K. pneumoniae* ST258 ^[21,22]^. Activation of IL-17 signaling is being explored as a vaccine strategy ^[23]^. Yet, despite the detailed analyses of how *K. pneumoniae* activates innate and adaptive immune responses, it has been difficult to understand why some strains evoke an immune response permissive of infection, whereas others cause a fulminant proinflammatory response.

## 3. Differences in the host response to *K. pneumoniae* pathotypes

The two major *K. pneumoniae* pathotypes activate distinct host defense mechanisms. The classical strains cause a subdued albeit progressive infection in various animal models, with the bacteria persisting even 10 days postinfection in the murine airways ^[24]^, or 13 days postinfection in a macaque pneumonia model ^[25]^. These observations are in line with the indolent infection seen in humans. Consistent with this more subacute infection, these bacteria elicit a myeloid response dominated by myeloid-derived suppressor cells (MDSCs) that fail to eliminate *K. pneumoniae* ^[11]^. This is accompanied by the accumulation of anti-inflammatory M2-like macrophages in the airway of the infected animals, along with anti-inflammatory mediators such as IL-10 and itaconate ^[12,24]^. The immune response to classical *K. pneumoniae* limits immunopathology and prioritizes host survival (Figure 1B) over the control of pathogen load, all features of host tolerance to infection (Figure 1C) ^[26]^.

In contrast, the hypervirulent strains, including KPPR1 (a rifampin-resistant ATCC 43816 derivative ^[27]^) that is commonly used in the laboratory, induce a rapidly lethal infection with the same bacterial inoculum (10^8^ CFUs). Even lower inocula (10^4^ CFUs) of hypervirulent *K. pneumoniae* are sufficient to cause lethality (Figure 1B) when introduced by the intranasal or intratracheal route in mice, orders of magnitude lower that the 50% lethal dose (LD_50_) of classical strains ^[15,28]^. This brisk inflammation with an influx of inflammatory monocytes and neutrophils constitutes a host response resistant to infection ^[26]^, which aims to limit the pathogen burden but is inadvertently associated with significant immunopathology and lethality ^[28]^ (Figure 1B,C). It is of note that metabolism is critical in determining the nature of the host defense (tolerance or resistance) to infection.

## 4. Immunometabolism

The specific metabolic pathways used to maintain the generation of energy (ATP) in immune cells are also important in defining their immune function. The concept of cellular metabolic plasticity was first introduced by Otto Warburg in the field of oncology. In many tumor settings, cancer cells shift from mitochondrial oxidative phosphorylation (OXPHOS) to glycolysis by increasing their consumption of glucose even under normoxia ^[29]^. Given that glycolysis is less efficient than OXPHOS in terms of energy production, this may appear counterintuitive. However, the sustained increase in the rate of glycolysis matches the anabolic needs of the rapidly dividing tumor cells. Ongoing studies have revealed that other metabolic activities, even those that fuel OXPHOS, are also upregulated in tumors ^[30]^. Thus, tumor cells sustain their proliferation and survival through diverse metabolic changes depending on the tumor microenvironment. These initial investigations paved the way to cancer immunotherapy, whereby metabolic pathways are targeted to eliminate cancer cells, a strategy that has proven to be very successful ^[31,32]^.

**Figure 2.**
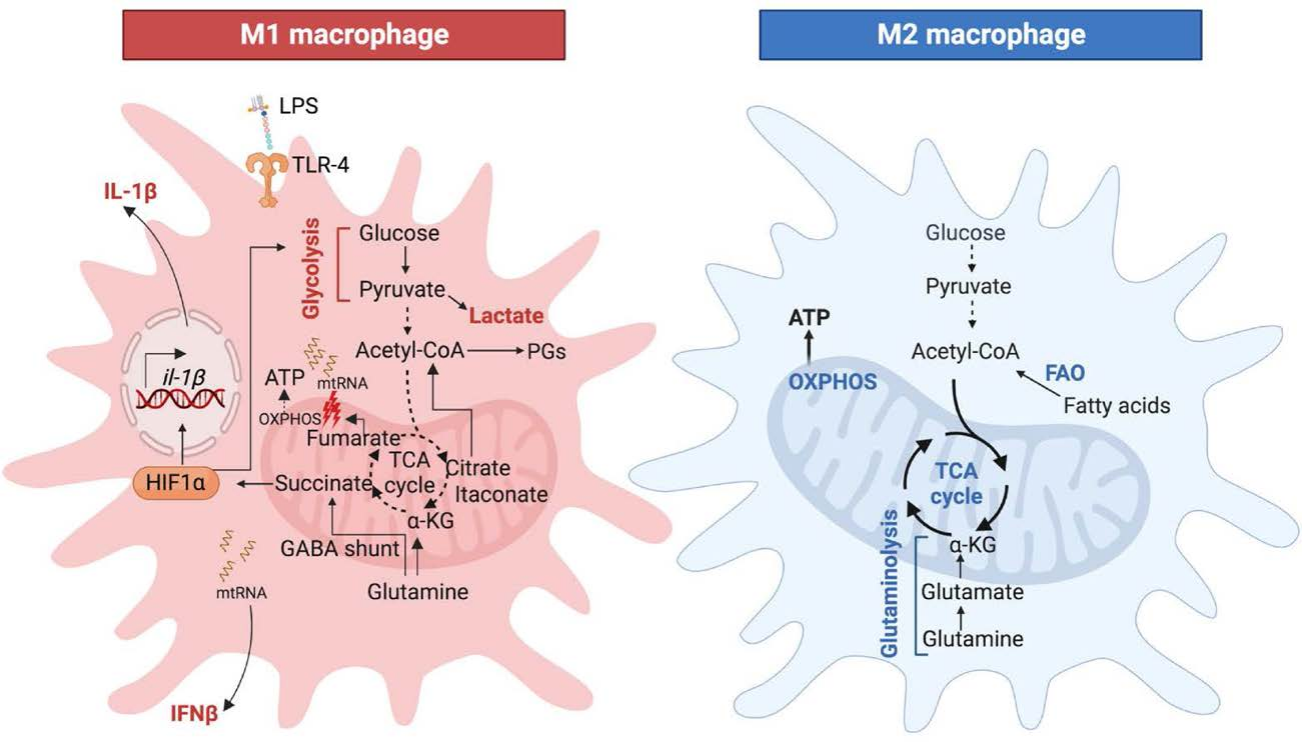
**Metabolic activities dictate the function of immune cells.** (Left panel) Inflammatory (M1) macrophages accumulate the TCA metabolites citrate, succinate, fumarate and itaconate as a result of several breaks in the TCA cycle. Succinate and citrate promote glycolysis and inflammation. Fumarate impairs OXPHOS through mitochondrial damage. The release of mitochondrial RNA (mtRNA) induces IFNβ production. Itaconate restores homeostasis via its anti-inflammatory and antioxidative properties. (Right panel) Anti-inflammatory (M2) macrophages downregulate glycolysis and upregulate metabolic pathways such as glutaminolysis and FAO that fuel the TCA cycle, OXPHOS and the production of reactive oxygen species (ROS).

Over the past decade, metabolic reprogramming has been revisited in the context of stimulation with the bacterial immunogen LPS ^[33]^. Several interruptions in the tricarboxylic acid cycle (TCA) are observed in LPS-stimulated macrophages. As a result, these macrophages accumulate the mitochondrial metabolites citrate, succinate, fumarate, and itaconate. Succinate is produced via glutamine-dependent anaplerosis or the γ-aminobutyric acid (GABA) shunt ^[10]^. Upon accumulation, succinate is exported from the mitochondria to the cytosol where it stabilizes the transcription factor hypoxia-induced factor 1 alpha (HIF-1α). HIF-1α in turn promotes glycolysis and the production of the proinflammatory cytokine IL-1β ^[10]^ (Figure 2, left panel). Concurrently, HIF-1α downregulates OXPHOS through the deregulation of pyruvate dehydrogenase kinase 1 ^[34]^, a phenotype that mirrors the Warburg effect.

Major proinflammatory activities of fumarate have been recently highlighted, focusing upon its role in type I interferon (IFN) activation ^[35]^. Fumarate accumulates in the cytoplasm of LPS-stimulated macrophages as a result of increased flux in the aspartate-argininosuccinate shunt as well as the inhibition of the enzyme fumarate hydratase. This causes mitochondrial stress and damage, impairing respiration and releasing mitochondrial RNA (mtRNA), which induces the expression of *Ifnb1* and the production of the cytokine IFNβ. Surprisingly, this was in sharp contrast to fumarate esters, which are successfully used in the management of inflammatory diseases such as psoriasis and multiple sclerosis ^[36]^. In addition, the inhibition of fumarate hydratase suppresses the expression of *Il-10* that leads to increased TNFα release ^[35]^.

Accumulated citrate, which is exported from the mitochondria to the cytoplasm, is converted to acetyl-coA that is directed towards the production of inflammatory lipid-mediators such as prostaglandins (PGs) ^[37]^ (Figure 2, left panel). The above-mentioned metabolic activities polarize the macrophage to a proinflammatory M1 phenotype. It should be noted that macrophage function is more complex than the convenient but overly simplified M1 (proinflammatory) and M2 (anti-inflammatory) classification, with a heterogeneous spectrum in between the two extremes. Following inflammation promoted by the metabolites citrate, succinate and fumarate, itaconate is produced in the mitochondrial matrix from citrate by the enzyme aconitate decarboxylase 1, Acod1 (also called Irg1). Itaconate exerts anti-inflammatory and antioxidative properties to restore homeostatic balance ^[38]^.

In contrast, M2-like macrophages upregulate OXPHOS through catabolic pathways such as fatty acid oxidation (FAO) and downregulate glycolysis ^[39,40]^ (Figure 2, right panel). Thus, the concept that metabolic activities dictate the function of immune cells was coined as “immunometabolism” in the early 2010s ^[41]^. Since then, several seminal studies have demonstrated how diverse metabolites function as signaling intermediates that dictate the immune response ^[10,35,42–44]^. Interestingly, it is increasingly being recognized that bacterial pathogens can subvert immunometabolism to promote infection ^[12,45,46]^.

## 5. Role of LPS in the immunometabolic response to *K. pneumoniae*

Many bacterial gene products are highly stimulatory, some more so than others. *K. pneumoniae* lacks some of the major immunostimulatory pathogen-associated molecular patterns expressed by other Gram-negative opportunists, such as flagella or the Type Three Secretion System (T3SS). Thus, LPS is the major proinflammatory PAMP produced by *K. pneumoniae*. Numerous studies established the importance of the LPS-TLR4-MyD88 signaling cascade in initiating the host inflammatory response to *K. pneumoniae* ^[47–49]^. The LPS of *K. pneumoniae* is structurally similar to prototypical LPS from *Escherichia coli* and as immunostimulatory ^[12]^. LPS stimulates resting macrophages, causing them to switch from their basal metabolic state (OXPHOS) to glycolysis, a metabolic change that fuels the downstream proinflammatory signaling. While this is entirely consistent with the brisk and robust inflammation that accompanies infection with the hypervirulent strains, it does not correlate with the subacute infection induced by the classical strains.

Modifications of *K. pneumoniae* LPS, especially in the lipid A moiety, were reported as a mechanism of immune evasion ^[50]^. However, a direct comparison of the LPS from a representative classical MDR *K. pneumoniae* strain (MKP103—a KPNIH1 derivative strain ^[51]^, ST258) and a representative hypervirulent strain (KPPR1, ST493) revealed only minor differences ^[12]^. The overall immune response to purified LPS from these two *K. pneumoniae* strains was comparable, with similar recruitment of innate immune cells and cytokine induction ^[12]^. Of note, LPS from both *K. pneumoniae* strains elicited the same metabolic response in the airway (ie, the airway metabolome) and were associated with a substantial release of the host anti-inflammatory metabolite itaconate ^[12]^. Thus, LPS by itself seems unlikely to account for the distinctive host immune defenses to the different *K. pneumoniae* pathotypes.

## 6. Live *K. pneumoniae* ST258 stimulates a different airway metabolic response than the hypervirulent strain

**Figure 3.**
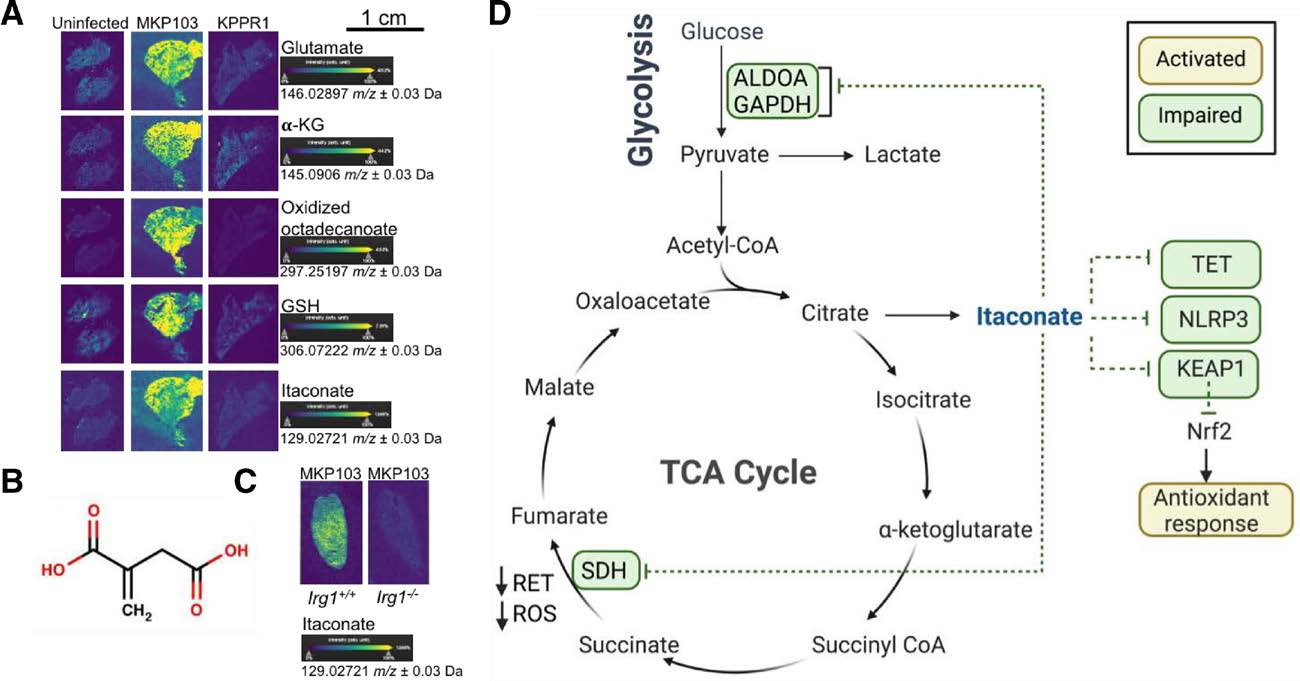
**Metabolic responses during *K. pneumoniae* infection.** (A) *K. pneumoniae* strains MKP103 and KPPR1, representative of classical and hypervirulent pathotypes, respectively, induce distinct metabolic responses in the lung of BL/6 mice, as demonstrated by mass spectrometry imaging (DESI-MS) ^[12]^. (B) Structure of the metabolite itaconate which (C) accumulates in the lung of *Irg1*^+/+^ (BL/6) but not *Irg1*^−/−^ control mice during *K. pneumoniae* infection. (D) Itaconate exerts anti-inflammatory and antioxidative effects in host cells via numerous mechanisms. (A) was reproduced with permission from ^[12]^, copyright © 2022 Elsevier.

Major differences were observed in the airway metabolic response to live bacteria from each pathotype (MKP103 and KPPR1), as contrasted to the similar responses to their purified LPS ^[12]^. Notably, these distinctive airway metabolic profiles were in response to equivalent bacterial burden in the mouse lung. Perhaps this should be expected as the metabolic properties of each of the representative strains themselves differ ^[12]^. The differences in airway metabolomes during infection with either pathotype was readily apparent using both liquid chromatography mass spectrometry (LC-MS) of murine bronchoalveolar lavage fluid (BALF) and desorption electrospray ionization mass spectrometry (DESI-MS) imaging of infected lung tissues ^[12]^ (Figure 3A). DESI-MS enables the in situ localization of metabolites of interest, a technique which has been harnessed in the field of oncology to discriminate between cancerous and adjacent normal tissues based on the spatial distributions of specific metabolites ^[52]^. While neither of the above approaches can distinguish bacterial from host metabolites during infection, they are complementary techniques that broadly detect the airway metabolome, particularly in infected versus uninfected animals.

Glutaminolysis (degradation of glutamine to glutamate and α-ketoglutarate) and FAO (breakdown of fatty acids to acetyl-coA) were induced in the airway of *K. pneumoniae* ST258-infected mice ^[12]^. These metabolic pathways were activated even when the animals were infected with bacterial mutants lacking these metabolic routes, indicating that they are predominantly associated with the host rather than the bacterial metabolism ^[12]^. Host glutaminolysis and FAO drive anaplerosis (replenishment of the TCA cycle intermediates) and OXPHOS, perhaps as a consequence of glucose depletion ^[12]^. These metabolic activities were not observed during either infection with the hypervirulent strain, KPPR1, or stimulation with purified LPS from *K. pneumoniae* or *E. coli*. The urea cycle, the metabolism of arginine, aspartate, and ketone bodies as well as the production of the antioxidants, glutathione and itaconate, were also significantly increased in murine lungs infected with classical *K. pneumoniae* ^[12]^. Interestingly, FAO and the production of antioxidative and anti-inflammatory metabolites were similarly induced during systemic infection in rats ^[53]^. Pharmacologic and genetic inhibition of FAO/glutaminolysis and itaconate production, respectively, resulted in heightened inflammation in the lungs of the infected mice and an acute infection ^[12]^. These findings demonstrate that FAO, glutaminolysis, and itaconate generate a metabolic milieu that supports or is at least tolerant of indolent infection. Thus, despite the immunostimulatory activity of LPS, classical strains appear to exhibit metabolic activity sufficient to override LPS signaling and drive a permissive immunometabolic response. More extensive characterization of the airway immunometabolic response to diverse *K. pneumoniae* strains from each pathotype are required to determine whether MKP103 and KPPR1 are broadly representative of the two pathotypes.

## 7. Immune signaling associated with fatty acid oxidation and glutaminolysis

The importance of FAO and glutaminolysis in directing the effector function of immune cells is well appreciated ^[39,40,54]^. Compared with the induction of inflammation by glycolysis, FAO, and glutaminolysis generally support anti-inflammatory signaling. M2-like macrophages stimulate FAO that fuels the TCA cycle and OXPHOS. Glutaminolysis also stimulates the polarization of M2-like macrophages through the production of α-ketoglutarate and its epigenetic effects ^[39,40]^. The trimethylation of lysine 27 in histone H3, H3K27me3, prevents the expression of M2 marker genes. α-ketoglutarate costimulates the enzyme Jumonji-domain containing 3, Jmjd3, which demethylates H3K27 on the promoters of M2-specific genes, relieving their repression ^[40]^. In addition, α-ketoglutarate inhibits proinflammatory NF-кB signaling and fuels FAO and OXPHOS. Glutamine also supports M2 polarization via the hexosamine biosynthetic pathway, whereby glutamine is directed toward the production of uridine diphosphate *N*-acetylglucosamine (UDP-GlcNAc) ^[39]^. UDP-GlcNAc is crucial for M2 polarization given that it glycosylates M2 protein markers ^[39]^. M2-like macrophages promote immunosuppression through their role in tissue repair and the resolution of inflammation by anti-inflammatory mediators such as arginase 1, IL-10, and TGF-β. Thus, the prominence of FAO and glutaminolysis in the metabolic response to classical *K. pneumoniae* would promote host tolerance to infection as opposed to the highly inflammatory signaling associated with glycolysis.

Increased fatty acid uptake and the induction of FAO are features of MDSCs, a heterogenous population of immature myeloid cells with immunosuppressive properties ^[54]^. These cells are best characterized for their role in promoting tumor progression. They inhibit T-cell activity and proliferation through the depletion of amino acids that are crucial for T-cell function (eg, arginine), or the production of reactive oxygen species (ROS), which block the T-cell receptor (TCR)-MHC-peptide interaction ^[55]^. Their immunosuppressive function is also associated with their production of the anti-inflammatory metabolite itaconate ^[56]^. Interestingly, myeloid cell-derived itaconate has recently been shown to enhance FAO in nonmyeloid cells (hepatocytes), amplifying its immunomodulatory signaling in trans ^[57]^. Pharmacologic inhibition of FAO using etomoxir hindered the immunosuppressive function of MDSCs in mouse tumor models and significantly delayed tumor growth in a T cell-dependent manner ^[58]^. When combined with low-dose chemotherapy, FAO inhibition completely abrogated the immunosuppressive effects of MDSCs, resulting in an enhanced antitumor response ^[58]^.

The stimulation of host FAO and glutaminolysis during *K. pneumoniae* ST258 infection is highly reminiscent of the induction of these OXPHOS-fueling (and ROS-generating) pathways in tumors and the accompanying accumulation of M2-like macrophages and MDSCs. Consistent with this local metabolic milieu is the relative enrichment of these immunosuppressive myeloid cells in response to *K. pneumoniae* ST258 infection, in contrast to the more glycolytic, proinflammatory M1-like macrophages that responded to stimulation with purified LPS ^[12]^. These differing myeloid cell populations may be important in promoting host tolerance to prolonged infection with classical *K. pneumoniae*.

## 8. Myeloid cell populations recruited by *K. pneumoniae*

Neutrophils and monocytes are among the major myeloid cells recruited to the lung during *K. pneumoniae* infection. Studies by DeLeo and coworkers indicated that despite the influx of neutrophils to sites of *K. pneumoniae* ST258 infection, the bacteria were not efficiently killed due to impaired phagocytosis ^[59]^. Numerous studies have indicated that hypervirulent *K. pneumoniae* strains also evade neutrophil phagocytosis ^[59]^, particularly owing to the presence of their thick capsule ^[60]^. Nonetheless, neutrophil depletion resulted in increased host mortality to hypervirulent *K. pneumoniae* as a result of high pathogen load ^[15,61]^, but not to *K. pneumoniae* ST258 ^[11]^. The monocyte/macrophage populations, in contrast, are extremely important in the clearance of *K. pneumoniae* strains from either pathotype ^[15,61]^. Depletion of CCR2^+^ inflammatory monocytes impaired clearance of different *K. pneumoniae* strains from the lung, as did the depletion of alveolar macrophages ^[15,61]^.

Discrete monocyte populations have been identified in response to the distinct *K. pneumoniae* infections. Over the past decade, the roles of MDSCs in both oncology and infectious diseases have been studied and specific nomenclature for these immature myeloid cells has altered as we learn more about them, especially the differences in the monocytic versus granulocytic subtypes ^[62,63]^. Some of the earlier studies of the MDSC population indicated these cells that are nonmigratory and do not traffic to the lymph nodes ^[64]^. Instead, they undergo a selective enrichment in the lung, early during infection with the classical *K. pneumoniae* strains ^[11]^. As a source of both itaconate and IL-10 ^[24,56]^, the MDSCs have the potential to shape a local immunometabolic milieu that is conducive to infection. Using a classical *K. pneumoniae* ST258 strain from a US hospital outbreak (KP35), Ahn and coworkers noted the abundance of the monocytic MDSCs (CD11b^+^Ly6C^+^Ly6G^lo^) rather than the granulocytic MDSCs (CD11b^+^Ly6C^lo^Ly6G^+^) early during infection (2 days) ^[11]^. This was recently confirmed by Wong *et al.* using single cell RNA-sequencing of the murine lung infected with another ST258 strain (MKP103) ^[12]^. These anti-inflammatory monocytic MDSCs were poorly phagocytic and impaired in their ability to kill the bacteria ^[11]^. The kinetics of MDSC recruitment seems to vary according to the type of *K. pneumoniae* infection. Studies by Poe et al, a decade ago, indicated that hypervirulent *K. pneumoniae* (ATCC 43816) infection elicited the influx of CD11b^+^Gr1^int^F4/80^+^ cells ^[65]^, phenotypically similar to MDSCs. However, these MDSCs were recruited later during infection with the hypervirulent strain (3 days) and were shown to mediate the resolution of acute pneumonia through the production of IL-10 ^[65]^. Attempts at selectively depleting MDSCs without affecting other myeloid cell populations have not been successful ^[11]^ as there have not been markers that exclusively discriminate MDSCs from other myeloid cells.

## 9. Itaconate contributes to subacute pulmonary infection by *K. pneumoniae* ST258

MDSCs and other myeloid cells produce the mitochondrial metabolite itaconate. Itaconate, a C5-dicarboxylate, was one of the most abundant metabolites found in the *K. pneumoniae*-infected airway, particularly in response to the classical ST258 strain ^[12]^ (Figure 3A–C). This immunometabolite is well known to be induced by LPS and to function as an anti-inflammatory and antioxidative agent to prevent host damage from inflammation via a number of mechanisms ^[38]^ (Figure 3D). Again following a parallel, in oncology, itaconate is associated with tumor persistence ^[56,66]^ through its multiple effects in suppressing immune activation. As a weak electrophile, itaconate exerts its immunomodulatory role by irreversibly modifying cysteine residues (nucleophiles) within its targets, a process called Michael addition, alkylation, dicarboxypropylation or itaconation. Alkylation of the NLRP3 inflammasome ^[43]^ and glycolytic enzymes such as glyceraldehyde-3-phosphate dehydrogenase (GAPDH) and fructose-bisphosphate aldolase A (ALDOA) ^[67]^, results in the inhibition of their enzymatic activity and the release of the proinflammatory cytokine IL-1β. The electrophilic properties of itaconate promote an antioxidative response through the alkylation of the redox-sensing protein Kelch-like ECH-associated protein 1 (KEAP1) ^[42]^. KEAP1 normally associates with the transcription factor, nuclear factor E2-related factor 2 (Nrf2), and promotes its degradation. Alkylation of crucial KEAP1 cysteine residues enables newly synthesized Nrf2 to accumulate, translocate to the nucleus and activate a transcriptional antioxidant and anti-inflammatory program ^[42]^. Importantly, despite sharing electrophilic properties and core mechanisms of action, the itaconate derivatives 4-octyl-itaconate (4-OI) and dimethyl-itaconate (DI), which were originally used as a proxy for itaconate because of their cell permeability, also exhibit distinct immunological effects ^[68]^. For example, 4-OI and DI suppress IFNβ production whereas itaconate promotes type I IFN signaling ^[68]^. This work led to itaconate being re-defined as immunoregulatory rather than strictly anti-inflammatory or immunosuppressive. The re-evaluation of itaconate and its derivatives revealed that 4-OI and DI are not metabolized to itaconate intracellularly, and that exogenous itaconate readily enters the cytosol of cells although it is not clear how.

Itaconate also inhibits succinate dehydrogenase (SDH)/mitochondrial complex II ^[44]^ and TET DNA dioxygenases ^[69]^. The inhibition of SDH by itaconate results in decreased mitochondrial ROS production through the reverse electron transport (RET) and increased succinate levels ^[44]^. This indicates that succinate oxidation rather than accumulation promotes mitochondrial ROS generation and proinflammatory signaling. Itaconate suppresses the expression of proinflammatory genes in both the STAT and NF-кB signaling pathways through the inhibition of TET DNA dioxygenases, enzymes that induce DNA demethylation ^[69]^. A novel post-translational modification has been attributed to a degradation product of itaconate, itaconyl-CoA. Itaconyl-CoA-mediated lysine acylation (called itaconylation) of histones suggests a new mechanism of epigenetic modulation by itaconate ^[70]^.

The production of itaconate by myeloid cells including macrophages, neutrophils and MDSCs is important for preserving tissue integrity and host health. As expected, the absence of itaconate in vivo (as in the *Irg1*^*−/−*^ mice, Figure 3C) promotes acute pulmonary infection especially by classical *K. pneumoniae* ^[12]^, presumably due to excess tissue damage and unhindered bacterial replication. While protecting the host, the buildup of itaconate may promote the persistence of classical *K. pneumoniae* by limiting inflammasome activity and the generation of ROS that are crucial for bacterial killing, especially in a setting in which neutrophil-mediated bacterial clearance is minimal at best. Interestingly, itaconate also drives bacterial persistence through its direct effects on the bacteria ^[71,72]^. Itaconate has previously been attributed bactericidal properties given that it alkylates and inhibits bacterial isocitrate lyase that is vital for bacterial survival via the glyoxylate shunt ^[71,72]^. As a result, the bacteria adapt to itaconate and persist by redirecting their metabolic activities toward biofilm production for protection ^[73,74]^.

## 10. Adaptation of MDR *K. pneumoniae* to the immunometabolic milieu includes the upregulation of the T6SS

**Figure 4.**
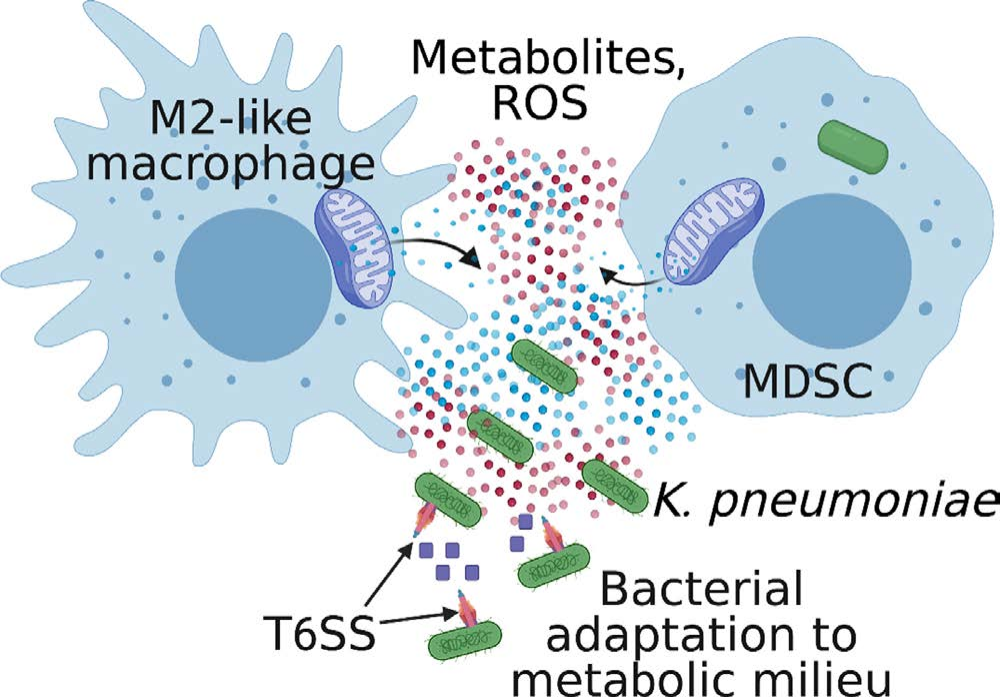
**Adaptation of *K. pneumoniae* to the airway metabolic milieu.** During infection with classical *K. pneumoniae*, immunosuppressive myeloid cells (M2-like macrophages and MDSCs) accumulate in the airway, producing reactive oxygen species (ROS) as a result of OXPHOS-fueling pathways. This metabolic milieu induces bacterial adaptation via the upregulation of the T6SS.

In addition to itaconate, other airway metabolites and reactive oxygen and nitrogen species generate selective pressure (immunometabolic pressure) on the bacteria. Successful pathogens rapidly change their transcriptional profile in response to local environmental conditions, which evolve over the course of infection as oxidants and many metabolic substrates accumulate in the lung. Both in vivo and in the presence of ROS in vitro*, K. pneumoniae* ST258 upregulates genes involved in redox homeostasis and those that encode the Type Six Secretion System (T6SS) ^[12]^ (Figure 4), which participates in the bacterial antioxidant response ^[12]^.

The T6SS consists of core components that are organized as the baseplate, membrane complex, tube, sheath, and spike ^[75]^. This phage-like apparatus delivers effector proteins directly into target cells (bacterial or eukaryotic) using a membrane-piercing spike ^[76]^. Several T6SS effectors (eg, catalases and zincophores from other bacterial species ^[77–79]^) are secreted in the extracellular milieu in a contact-independent manner to counteract environmental stresses ^[80]^. While the T6SS is best characterized for its role in killing non-kin bacterial competitors, its role in mounting an antioxidant response to mediate bacterial survival may be more relevant in the airway than interbacterial killing given the sparse lung microbiome ^[81]^. Indeed, the T6SS was demonstrated to be vital for *K. pneumoniae* survival under excessive oxidant stress in vivo ^[12]^, although the mechanism of action is yet to be ascertained. This raises the possibility of T6SS effectors that promote bacterial adaptation to the airway metabolic milieu. Interestingly, a T6SS effector of *K. pneumoniae*, VgrG4, has recently been shown to target the mitochondria ^[82]^, raising yet another possibility of direct host metabolic subversion by the T6SS.

## 11. Metabolic adaptation of MDR *K. pneumoniae* to the airway milieu

The genetic flexibility of the classical *K. pneumoniae* strains is well illustrated. For example, KP35, an ST258 strain from our hospital, was noted to have acquired 4 novel open reading frames (ORFs) compared to *K. pneumoniae* strains from other STs ^[11,83]^. Subsequent studies demonstrated that one of these ORFs encoded an acyl transferase (*atf3*, previously annotated as *arcD*), associated with the acetylation of a substantial number of metabolic enzymes ^[83]^. Atf3 drove the enhanced metabolic fitness of KP35 in a mouse model of pneumonia ^[83]^. Thus, the acquisition of an ORF that enhances *K. pneumoniae* metabolic activity promotes the bioenergetics of the pathogen and its success in the airway milieu.

## 12. Conclusions

*K. pneumoniae* is a tremendously successful human pathogen in part due to its metabolic versatility, which not only enhances its fitness but also enables it to shape the metabolic response to infection and ultimately the infection outcome. Live classical *K. pneumoniae* induces a distinct airway metabolome compared with either the heat-killed bacteria or live hypervirulent *K. pneumoniae*. This metabolome translates into an immunosuppressive response, with M2-like macrophages and MDSCs predominating instead of the more proinflammatory M1-like macrophages expected to clear a typical Gram-negative infection.

The metabolic potential of *K. pneumoniae* has long been recognized and considered a target for therapy ^[84]^. However, these more recent studies documenting the impact of bacterial metabolic activity in shaping the immune response to in vivo infection ^[12,45,74,85]^ suggest that *K. pneumoniae* metabolism itself is a major factor in pathogenesis. This was perhaps to be expected as not all *K. pneumoniae* strains share the same metabolic potential. Various *K. pneumoniae* lineages have different core metabolic capabilities ^[86]^. The ability of *K. pneumoniae* ST258 to impose such a profound metabolic stress on the host as to override the macrophage reprogramming associated with LPS, functions as a virulence strategy. This enables the bacteria to persist in the lung while suppressing host damage that would also be detrimental to the bacteria. However, there remains a possibility that other virulence strategies associated with live bacteria may play a role in the immunometabolic subversion by the pathogen. These include products secreted by live bacteria such as T6SS effectors that have not yet been identified and characterized. Furthermore, the timing of the metabolic subversion seems to be important in determining the nature of the host immune response and the infection outcome, adding another layer of complexity to this dynamic host-pathogen interaction.

In more recent years, the distinction between the two *K. pneumoniae* pathotypes has become less evident, particularly with the emergence of strains that are both hypervirulent and drug resistant ^[6,87]^. Whilst these strains are of major concern, whether they will persist in the environment or in patients is still under epidemiological surveillance and clinical investigation ^[6,88–90]^, especially given that the acquisition of either virulence genes by classical strains or multidrug-resistance genes by hypervirulent strains is expected to come at a fitness cost. If these strains do persist, their metabolism as well as the type of immunometabolic response they induce will need to be thoroughly characterized. Further studies will reveal how host-based strategies centered on immunometabolic manipulation may inadvertently drive bacterial adaptation. This is an important aspect for consideration in order to avoid repeating past mistakes such as the development of resistance mechanisms as a result of high selective pressures. More work is required to understand how *K. pneumoniae* adapts to immunometabolites.

Regardless, the cross-talk between bacterial pathogens and the host should be further explored considering how metabolic manipulation has proven to be a success in the treatment of cancers, the epitome of immunometabolic subversion. The application of immunometabolic therapy to clear multidrug-resistant bacteria such as *K. pneumoniae*, holds great promise, as many metabolic inhibitors have already been FDA-approved and utilized.

## Author contributions

Both authors have made a substantial, direct and intellectual contribution to the manuscript and have approved it for publication.

## Conflicts of interest

The authors declare that this work was conducted in the absence of any commercial or financial relationships that could be construed as potential conflicts of interest.

## Funding

AP was supported by NIH grant R35 HL135800 and the Cystic Fibrosis Foundation CFF PRINCE18G0. TWFL was supported by NIH grant K99 HL157550.

## Acknowledgments

Figures were created with Biorender.com.
